# Supplementary material for: Splice-Junction-Based Mapping of Alternative Isoforms in the Human Proteome
Source: Cell Rep. Author manuscript; Available in PMC 2020 Jan 15. (PMC6961840; doi:10.1016/j.celrep.2019.11.026)
Supplement: 2 [file NIHMS1546469-supplement-2.zip › DF1/PXD006675/LeftCoronaryArtery/LeftCoronaryArtery_11_ABCA2_GHLPATALR.pdf]

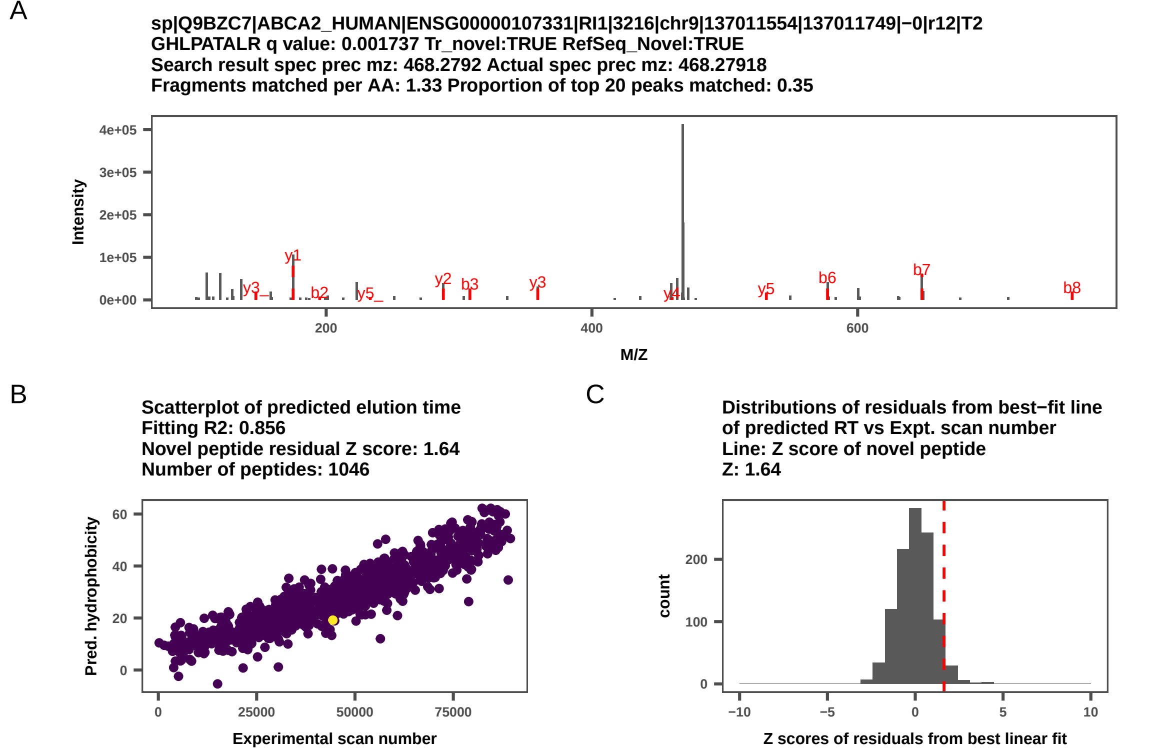
A
sp|Q9BZC7|ABCA2_HUMAN|ENSG00000107331|RI1|3216|chr9|137011554|137011749|−0|r12|T2
GHLPATALR q value: 0.001737 Tr_novel:TRUE RefSeq_Novel:TRUE
Search result spec prec mz: 468.2792 Actual spec prec mz: 468.27918
Fragments matched per AA: 1.33 Proportion of top 20 peaks matched: 0.35
Intensity
4e+05
3e+05
2e+05
1e+05
0e+00
y1
y3
b2
y5
y2
b3
y3
y4
y5
b6
b7
b8
200
400
600
M/Z
B
Scatterplot of predicted elution time
Fitting R2: 0.856
Novel peptide residual Z score: 1.64
Number of peptides: 1046
Pred. hydrophobicity
60
40
20
0
0
25000
50000
75000
Experimental scan number
C
Distributions of residuals from best−fit line
of predicted RT vs Expt. scan number
Line: Z score of novel peptide
Z: 1.64
count
200
100
0
−10
−5
0
5
10
Z scores of residuals from best linear fit
